# Electrophysiological Correlates of Error Monitoring and Feedback Processing in Second Language Learning

**DOI:** 10.3389/fnhum.2017.00029

**Published:** 2017-01-30

**Authors:** Sybrine Bultena, Claudia Danielmeier, Harold Bekkering, Kristin Lemhöfer

**Affiliations:** ^1^Donders Institute for Brain, Cognition and Behaviour, Radboud UniversityNijmegen, Netherlands; ^2^School of Psychology, University of NottinghamNottingham, UK

**Keywords:** L2 learning, error monitoring, feedback processing, grammatical gender, ERN, CRN, FRN, P300

## Abstract

Humans monitor their behavior to optimize performance, which presumably relies on stable representations of correct responses. During second language (L2) learning, however, stable representations have yet to be formed while knowledge of the first language (L1) can interfere with learning, which in some cases results in persistent errors. In order to examine how correct L2 representations are stabilized, this study examined performance monitoring in the learning process of second language learners for a feature that conflicts with their first language. Using EEG, we investigated if L2 learners in a feedback-guided word gender assignment task showed signs of error detection in the form of an error-related negativity (ERN) before and after receiving feedback, and how feedback is processed. The results indicated that initially, response-locked negativities for correct (CRN) and incorrect (ERN) responses were of similar size, showing a lack of internal error detection when L2 representations are unstable. As behavioral performance improved following feedback, the ERN became larger than the CRN, pointing to the first signs of successful error detection. Additionally, we observed a second negativity following the ERN/CRN components, the amplitude of which followed a similar pattern as the previous negativities. Feedback-locked data indicated robust FRN and P300 effects in response to negative feedback across different rounds, demonstrating that feedback remained important in order to update memory representations during learning. We thus show that initially, L2 representations may often not be stable enough to warrant successful error monitoring, but can be stabilized through repeated feedback, which means that the brain is able to overcome L1 interference, and can learn to detect errors internally after a short training session. The results contribute a different perspective to the discussion on changes in ERN and FRN components in relation to learning, by extending the investigation of these effects to the language learning domain. Furthermore, these findings provide a further characterization of the online learning process of L2 learners.

## Introduction

Second language (L2) learners are prone to making grammatical errors, for example, those that originate from incompatibilities between the L2 and the learners' native language (L1). The L1 influences L2 processing as evidenced by transfer (Odlin, 1989, 2003; Ellis, 2006; Pajak et al., 2016), which can lead to persistent grammatical instability and mistakes even in experienced L2 speakers (White, 2003). To improve L2 proficiency and reduce mistakes, the cognitive system that deals with (internal) error monitoring and (external) feedback processing must play an important role. Such performance monitoring has largely been investigated for lower-level cognitive tasks (see Ullsperger et al., 2014), where mistakes arise from a temporal perceptual failure or erroneous action selection, and can often be detected by the participant immediately after response execution. In contrast, syntactic L2 errors frequently seem to arise from a failure to remember the correct form above the old, incorrect (L1-driven) form, even though it must have been repeatedly encountered in natural L2 input. This suggests that memory representations underlying L2 syntactic processing are unstable and subject to L1 interference, but there is little evidence from performance monitoring measures during L2 learning to support this idea. Using electrophysiological markers of error monitoring, the present study sets out to investigate the process of syntactic L2 learning by looking at neural correlates of error monitoring and feedback processing, to examine whether L2 speakers can detect their own errors at all, and how the success of error detection develops across a training session. Additionally, we look at neural processing of the feedback that leads to learning and memory stabilization (i.e., how stable representations are formed). Before introducing the present study, we will first discuss neural correlates relevant for internal and external monitoring, and signatures of learning.

### ERP correlates

#### Internal monitoring

In many day-to-day activities, people monitor their own behavior to identify and correct errors internally. When performance goes awry and anticipated goals are missed, the brain's performance monitoring system generates a characteristic event-related potential known as the error-related negativity (ERN) (Gehring et al., 1993) or error negativity (Ne) (Falkenstein et al., 1990). The ERN is a negative-going ERP wave time-locked to (incorrect) responses at the fronto-central midline, which peaks between 0 and 100 ms after an error has been committed (Gehring et al., 2011). The effect is typically observed in the context of speeded decision tasks that involve a response based on a perceptual decision, such as the Flanker task or anti-saccade task (Hohnsbein et al., 1991; Nieuwenhuis et al., 2001; Endrass et al., 2005; Danielmeier et al., 2009). Here, slips occur due to incomplete information accumulation, which can often be detected by the participant soon after the response (see Dambacher and Hübner, 2015). Usually, errors in such tasks are therefore accompanied by faster response times than in correct trials, while responses following errors are characterized by a slowdown of response times, known as post-error slowing (Rabbit, 1966; Danielmeier and Ullsperger, 2011). A number of studies has also shown a negatively peaking neural response for correct responses (see Vidal et al., 2000, 2003; Coles et al., 2001), which is known as the Correct Related Negativity (CRN) and has been linked to response uncertainty (Pailing and Segalowitz, 2004).

Typically, in experimental studies investigating the neural correlates of error monitoring, the errors involved concern performance errors or slips. Another common, but higher-level source of errors, known as mistakes (Wickens, 1992)^1^, however, occurs in learning situations that are less often investigated in the context of error monitoring (e.g., Hammer et al., 2013). In these situations, a correct response cannot be purely based on perceptual input, but involves a more complex mental operation (e.g., an arithmetic computation) or the retrieval of a memory representation. Previous studies examining memory retrieval after learning indicate the occurrence of an ERN effect for errors committed in a test phase following learning, although the size of the effect seems to depend on whether learning took place under errorful (in the context of competing incorrect responses) or errorless (without competing response alternatives) conditions (Rodriguez-Fornells et al., 2004; Heldmann et al., 2008a; Hammer et al., 2013). Errors in errorful conditions are characterized by a smaller ERN effect, because competing memory representations seem to complicate the internal detection of errors.

#### External monitoring

Errors pointed out by an external source of information yield a Feedback Related Negativity in the EEG (FRN; for an overview, see Luft, 2014), a component that is often linked to reinforcement learning (Holroyd and Coles, 2002). The FRN is a negative deflection on fronto-central electrodes that peaks around 200–300 ms following unexpected (i.e., informative) feedback (San Martín, 2012). Similar to the ERN, the neural response thus reflects the evaluation of an expected vs. an actual outcome (see Ernst and Steinhauser, 2012). This usually implies that negative feedback elicits a more negative signal than positive feedback, although the resulting signal is also determined by reward magnitude and likelihood of the outcome (Sambrook and Goslin, 2015).

The FRN usually co-occurs with a P300 effect that follows or is superimposed on the FRN (San Martín, 2012). The P300 effect is typically found in the centro-parietal region (surrounding CPz), although it is often visible across large parts of the head. Its timing ranges from 300 to approximately 600 ms post-feedback. In the literature, this ERP component is linked to memory updating; it signals the need for attention to adjust performance in order to bring about learning (Donchin and Coles, 1988; Ernst and Steinhauser, 2012). Furthermore, it is known to be inversely related to stimulus probability (Johnson, 1986), meaning that less frequent stimuli generate larger P300 effects.

Apart from showing a difference between positive and negative feedback, feedback-locked signals may also predict to what extent learning is taking place. A number of studies have shown that the size of the FRN following negative feedback is larger when behavior is correctly adjusted in the next trial compared to when no change in behavior is seen (e.g., Luu et al., 2003; Cohen and Ranganath, 2007; see San Martín, 2012, for a review), meaning that behavioral adaptation can be predicted based on the neural signal reflecting feedback processing. Other studies, however, only found differences in the P300 time window, as a reflection of memory processing in relation to subsequent behavioral adjustments (Yeung and Sanfey, 2004; Ernst and Steinhauser, 2012).

#### Changes in neural signatures during learning

Generally, the ERN can be considered a sign of the ability to monitor one's actions, but presumably only when there is little uncertainty about what the correct action is (i.e., after learning). In contrast, the FRN mostly arises when error monitoring still depends on external feedback (i.e., before learning has taken place). The learning process can thus be understood as a transition from relying on external feedback to developing an internal representation based on which one can monitor one's own performance. This is typically characterized by an inverse relationship between ERN and FRN components (Bellebaum and Daum, 2008; Heldmann et al., 2008b). When feedback becomes redundant, the size of the FRN decreases, as the external error signal becomes less surprising (Holroyd and Coles, 2002). Synchronously, the size of the ERN increases, indicating the correct response is known and the brain is able to detect errors independently. However, when experimental conditions do not allow for any learning to take place, the FRN remains large and no ERN is present (Eppinger et al., 2008; Glienke et al., 2015). Because internal error detection is only possible when correct representations are available, it is worthwhile to examine how such correct representations are formed. The L2 learning process seems a perfect example of incomplete learning, yet L2 learning has rarely been investigated from this perspective.

The learning process during L2 acquisition appears very suitable for examining how error monitoring develops as stability of long-term memory representations increases, and how feedback processing is related to successful learning. Presumably, during L2 learning, a stable correct representation must be formed in order for self-monitoring to function optimally. Previous studies have shown that ERP indicators of error monitoring are either absent or very small in L2 processing (Sebastian-Gallés et al., 2006; Davidson and Indefrey, 2011). Sebastian-Gallés et al. (2006) showed that L2 speakers fail to detect their own errors because of an influence of the L1. They tested Spanish learners of Catalan in an auditory lexical decision task. Crucially, in the critical condition, the word/non-word distinction in the task was based on a difficult Catalan sound contrast that does not exist in Spanish. As expected, the L2 learners of Catalan, who had difficulty to perceptually perceive this contrast and thus could not distinguish between pairs of words and non-words based on this contrast, did not show an ERN for their erroneous responses, while such an ERN was present for the errors made by Catalan L1 speakers. This suggests that error monitoring does not function optimally in L2 processing when mental representations do not allow to make a distinction between correct and incorrect responses.

Davidson and Indefrey (2011; see also 2009) more specifically looked at error monitoring at initial stages of learning. In their study, Dutch L1 speakers were asked to perform a non-speeded grammaticality judgment task based on phrases containing declension and gender violations in German, which are considered difficult for L2 speakers. The learners in this study, whose German proficiency was relatively low, received feedback by means of a color cue that allowed them to learn the grammatical rules. This study provided no evidence for successful self-monitoring in individual rounds of feedback, but a small ERN effect was present when response-locked data for the last two rounds were combined. The low proficiency of the participants in this task, and the fact that they were not actively learning and using German outside the context of the experiment, may have been reasons for this small effect.

In order to examine how processes of internal and external monitoring are related, we again turned to L2 learners. In contrast to the study by Davidson and Indefrey (2011), we examined relatively proficient immersed L2 learners and focussed on a feature that often presents difficulty to them in daily life because of an incongruence with their L1. The few existing studies on L2 learning indicate that it is possible to examine the learning process in the context of an experiment (e.g., Opitz et al., 2011) and that signs of learning can be seen before learning is reflected in behavior (McLaughlin et al., 2004), making this an interesting case to study behavioral adaptation and neural precursors thereof.

### The present study

In this study, we investigate error monitoring in relation to learning. In line with recent accounts that argue in favor of testing performance monitoring in more realistic situations (Wessel, 2014; Littman, 2015), we investigate monitoring in syntactic L2 learning as a case of higher order cognition. L2 learning presents itself as an interesting case, because this kind of learning also takes place outside experimental situations, thus implying a long-term goal, and involves stabilization of memory representations.

More specifically, we investigate how L2 learners form correct L2 representations that allow for successful error detection by studying the neural correlates of error monitoring and feedback processing in German learners of Dutch. This population is known to display frequent systematic errors in Dutch word gender, expressed in the use of determiners before nouns, that originate from incorrect L1 transfer (Lemhöfer et al., 2008, 2010). Most of these errors arise for translation equivalents that are similar in form, known as cognates. For these words especially, German learners of Dutch tend to associate German feminine and masculine gender to Dutch common gender (definite article “de”), and German neuter to Dutch neuter gender (definite article “het”). Hence, they derive at a “default” intuition for the grammatical gender of Dutch (in particular, cognate) nouns that is based on the gender of its German translation. This is a successful strategy for the majority of cognates, but gives rise to systematic errors on a number of nouns for which word gender in the two languages happens to be incongruent. In many cases, incongruent cognates, such as Dutch “*de* auto” (the car, common gender), will therefore incorrectly be produced as “*het* auto” (the car, neuter gender) by German learners of Dutch, because of the German translation “das Auto” (the car, neuter gender). Given that these errors are quite persistent, they are more likely to be representational than processing based (see Lemhöfer et al., 2014). In order to examine learning, we tested performance on these cognate nouns with incongruent gender by means of a gender assignment task with manual responses, involving three rounds with feedback. Participants were presented with word items (e.g., “auto,” car) and were required to press one button if they thought the common gender article “de” and the other button if they thought neuter gender article “het” was the correct definite article for this noun.

During learning, we looked at the ERN effect as a reflection of error monitoring, in combination with the FRN and P300 effects as correlates of feedback processing. Because there is some indication based on a number of studies that it is possible to predict learning based on how feedback is processed (Cohen and Ranganath, 2007; San Martín, 2012), we intended to see if this also applies to the L2 learning situation. In this context, we asked the following questions:

1. Are L2 representations of relatively proficient learners, especially those that are highly difficult (because of L1-L2 incongruence), strong enough to warrant successful error detection as reflected by the ERN?
2. If monitoring is initially absent, can sufficiently stable correct L2 representations be developed within the course of a restricted training session, leading to effective error monitoring toward the end of the session as indicated by the ERN?
3. How is feedback processed and does the size of neural correlates of feedback processing (FRN, P300) predict the incidence of learning?

With regard to the detection of erroneous responses, three scenarios were possible. A first possibility was that no difference in response-locked signals (ERN and CRN) would be observed between correct and incorrect responses. This was based on the notion that errors cannot be detected internally with sufficient certainty at the time of response as long as stable representations are not available yet. A second possibility was that errors would yield an ERN in comparison to correct responses, based on a mismatch between intended correct representations and actual incorrect responses. Apart from these possibilities, we considered a third alternative, namely that *correct* responses might show a larger response-locked negativity than errors. Given that our examined target nouns actually possess conflicting gender in Dutch and German (“de auto”), such a correct response would be “wrong” according to the learners' L1 representation. This reversal of the canonical ERN would be in line with findings by Lemhöfer et al. (2014), showing reversed P600 effects for correct determiner-noun phrases in cases where L2 learners had incorrect gender representations of the nouns.

Because we hypothesized that behavioral learning following feedback should be accompanied by internal detection of errors reflected by an ERN effect, we expected these different scenarios to occur at different stages of the experiment. In the initial stage, before learners had received any feedback or had fully acquired the correct representations of words, the absence of an ERN effect (no difference between error and correct conditions) or a reverse ERN effect were deemed most likely. We note that the absence of an effect could either indicate that learning had not yet taken place, or that multiple scenarios were at play at the same time such that opposite effects were canceled out. Some correct internal error detection was expected to occur for items that had been acquired before the experiment, which is not unlikely for fairly proficient L2 learners, who were immersed in a context in which they should have been exposed to the correct form repeatedly. In the course of the training task, following one or more rounds of feedback, we expected to see an overall trend toward canonical error detection, resulting in a normal ERN effect as an indication of learning.

Regarding feedback processing, we expected that FRN and P300 effects to negative feedback should initially be large in case stable internal representations were lacking. If feedback helps to overcome persistent errors, behavioral learning in the course of the experiment should be accompanied by fewer instances of negative feedback. Because the task involves learning on an item-by-item rather than rule basis, remaining instances of negative feedback should continue to be surprising for items that have not been learnt (remembered) yet. This implies that the FRN and P300 components would be visible in all subsequent rounds (see Eppinger et al., 2008; Glienke et al., 2015). Yet, repeated negative feedback was hypothesized to be less surprising, as indicated by reduced effects in the feedback-locked signal.

In addition to the main research questions, we also considered individual differences in learning. Learning accounts in the L2 acquisition domain stress the importance of differences among learners (e.g., Ehrman et al., 2003). Likewise, studies on performance monitoring suggest that characteristics of error detection are associated with aspects relevant to the learning process. Several studies examining the ERN have indicated that the size of the ERN depends on how serious the error is perceived to be, which in turn varies with individual differences in relation to anxiety (Frank et al., 2007; Hajcak, 2012), with certain and aware errors and errors with serious consequences yielding larger ERN effects. It is therefore conceivable that individual differences will play a large role in the way L2 learners monitor their performance. For that reason, we also collected scores on L2 proficiency and learning motivation, as well as certainty ratings in order to account for possible individual differences among L2 learners with regard to ERP effects.

## Materials and methods

### Participants

Twenty-six neurologically and psychiatrically healthy German learners of Dutch took part in the experiment after signing informed consent. Participants were recruited by means of flyers distributed on campus and messages send round to subjects registered in the Radboud University participant pool. All communication prior to testing was done in Dutch. Participants received monetary compensation (€10 per hour) in the form of gift vouchers or course credit in return for participation. One participant had to be excluded because of technical problems during recording, and another one because of a Dutch parent. One further participant had to be excluded because of too few errors (determined to be minimally 5 trials per round). This left data of 23 participants for analysis (5 male, 18 female; mean age 24 years; *SD* = 4; range: 19–39), all of whom were native speakers of German who had been speaking Dutch as a second language for minimally 1 year. All participants were right-handed according to an abridged version of the Oldfield handedness questionnaire and had normal or corrected-to-normal vision. The results of a language background questionnaire indicated that a majority of them (*N* = 20) resided in the Netherlands, and had lived there between 1.5 months and 16 years (*M*: 3.7 years; *SD* = 4) at the time of testing. Most of them had started to learn Dutch with the purpose of studying in the Netherlands. Participants also completed the Dutch version of the LexTALE vocabulary size test (Lemhöfer and Broersma, 2012; www.lextale.com) and a motivation questionnaire (see Procedure and Appendix A in Supplementary Material). All behavioral measures of L2 proficiency, use and motivation to learn the language are given in Table 1.

**Table 1:** **Means and standard deviations regarding proficiency, motivation and use of L2 Dutch (*N* = 23)**.

	**Mean**	***SD***	**Range**
LexTALE score	72	10	51–87
Dutch age of acquisition	20	2	13–24
Years of experience learning Dutch	4.5	4	1–17
Self-rated reading frequency	5.4	1.5	2–7
Self-rated speaking frequency	5.8	1.0	3–7
Self-rated listening frequency	6.2	0.8	5–7
Self-rated speaking proficiency	5.1	1.2	2–7
Self-rated listening proficiency	5.6	1.3	2–7
Self-rated writing proficiency	4.9	1.4	2–7
Self-rated reading proficiency	5.9	0.8	4–7
Self-rated overall proficiency	5.1	1.2	3–7
Motivation: General	18	2	13–20
Motivation: Perfectionism	17	2	13–20
Motivation: Perseverance	13	3	9–18
Motivation: Confidence	17	2	11–20

*LexTALE (Lexical Test for Advanced Learners of English) score is an averaged percentage correct over word and non-word items. Frequency and proficiency ratings were based on a 7-point scale ranging from 1 (low) to 7 (high). Scores for motivation, perfectionism, perseverance and confidence reflect summated scores across four questions per dimension based on 5-point scales (max 20 points per dimension)*.

### Stimulus material

Forty-eight Dutch nouns with a gender-incongruent cognate translation in German were selected as target stimuli for the gender decision experiment, which had been shown to be error-prone in previous studies (Lemhöfer et al., 2010; see also Lemhöfer et al., 2014). An additional 48 nouns were selected as fillers, which included 16 gender-congruent cognates, 16 incongruent non-cognates and 16 congruent non-cognates (see Appendix B in Supplementary Material for overview). The sole purpose of the fillers was to balance the stimuli in terms of cognate status and cross-linguistic gender congruence with respect to the gender response. All words were depictable Dutch singular nouns in their non-diminutive form; occurrences of “de” (common gender) and “het” (neuter gender) words were equiprobable in all conditions, but these categories were collapsed for analyses. Targets and fillers were matched on word length in letters (*M*_target_ = 5.6; *SD* = 1.4; *M*_filler_ = 5.6, *SD* = 1.6) and SUBTLEX log word form frequency (*M*_target_ = 2.7; *SD* = 0.7; *M*_filler_ = 2.7, *SD* = 0.6) in Dutch (Brysbaert and New, 2009). For each of the 96 items, a full color picture of an object against a white background was selected from the internet; all images were freely available for downloading. Pictures were resized to meet maximal dimensions of 180 by 180 pixels. An additional 18 words and matching pictures were used as practice items.

### Procedure

Participants were comfortably seated approximately 50 cm away from a computer monitor (1920^*^1080 resolution; 120 Hz refresh rate). An in-house designed button box, based on the BITSI (BIts To Serial Interface) protocol (see http://tsgdoc.socsci.ru.nl), with four buttons was placed on a table in front of them, of which two adjacent buttons were used for “de” and “het” responses that participants were requested to press with their left and right index fingers. During preparations for the EEG experiment, participants filled in the language background and handedness questionnaires. All communication between the experimenter and participant was done in Dutch.

The experiment started with a familiarization phase during which participants were tested on their knowledge of the 114 noun items featured in the experiment (96 experimental and 18 practice items). They were presented with each picture on the screen and asked to name the depicted object without using a determiner. In case of an incorrect response or omission, they received verbal feedback from the researcher. After every response, participants saw the correct word on the screen together with the picture.

**Figure 1:**
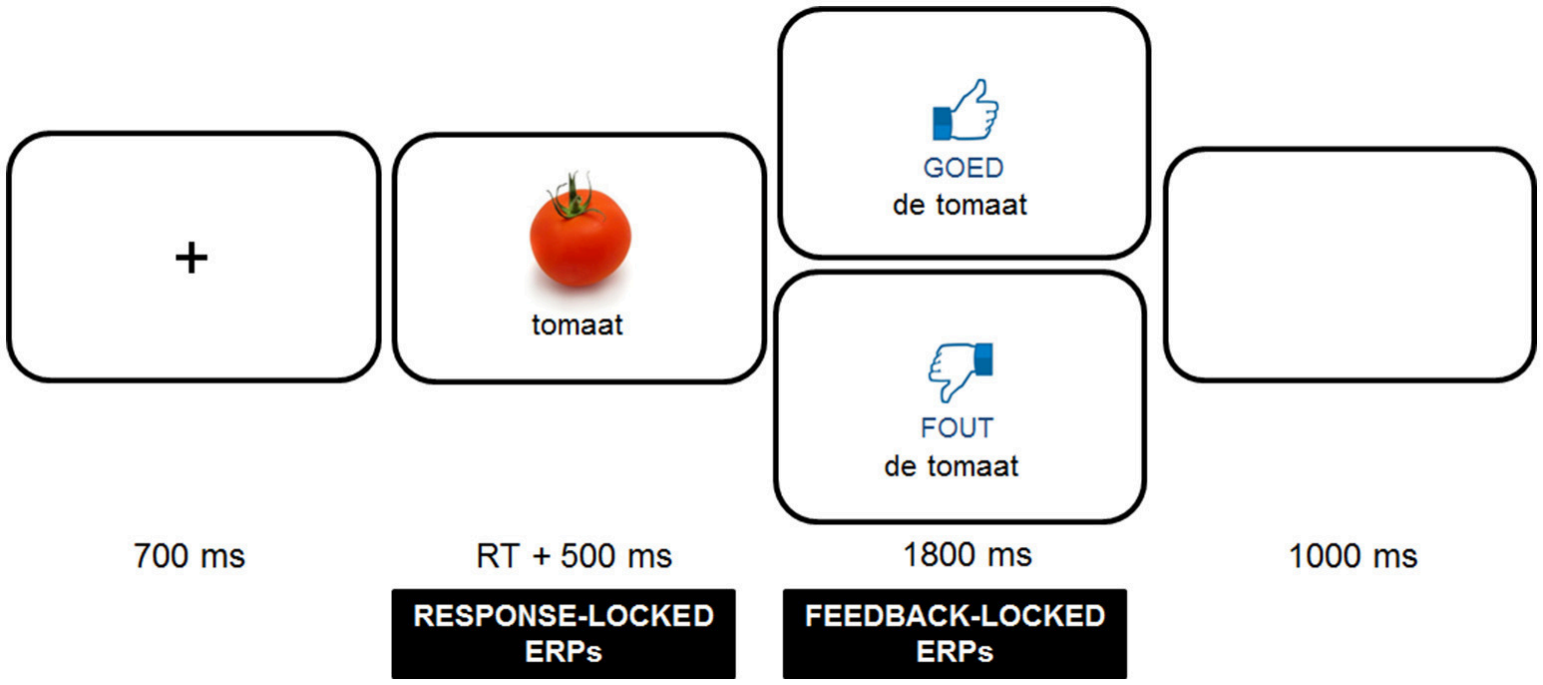
**Trial sequence used in the experiment**. Responses were followed by positive (“goed”) or negative (“fout”) feedback depending on response accuracy. Photo credits: FreeImages.com/Jean Scheijen.

After the familiarization phase, participants were presented with the gender decision task, which consisted of three rounds, preceded by a practice session (18 items). During each round, participants saw the 96 stimuli, consisting of a word and matching picture presented in the middle of the screen (approximately a 4 degree viewing angle), and had to decide on the appropriate definite determiner (“de” or “het”) by means of a button press (left button for “de” and right button for “het”). A trial always started with a fixation cross for 700 ms, followed by the stimulus, which stayed on the screen until participants had pressed a button. After the button press, the stimulus remained on the screen for 500 ms, before the participants received corrective feedback on the screen, which disappeared after 1800 ms. The feedback screen was made up of a thumbs up or thumbs down graphic presented in combination with the word “goed” (good) or “fout” (wrong) printed in black underneath, which was presented above the noun with its correct determiner. Before the next fixation cross appeared to signal the start of a new trial, participants had 1000 ms to blink (see Figure 1). Before testing, participants were verbally instructed to try and avoid blinking heavily during trials, but it was stressed that, if needed, it was better to blink occasionally than to avoid it. Four different pseudo-randomizations were created for the presentation of the stimuli using Mix (van Casteren and Davis, 2006), based on restrictions with regard to the determiner, cognate status and gender congruence (maximally 3 of each in a row). Furthermore, we ensured that adjacent items were never of the same semantic category and that there was no overlap in the last 15 items of a round and the first 15 items of the next round. After each round, participants received feedback on their performance presented as a percentage of correct responses and were encouraged to improve their performance in the next round. In the middle of and after every round, participants could take a small break. The first four items after a break were fillers. In total, the practice session and three rounds took approximately 30 min to complete.

After completion of the experiment, participants were given a paper and pencil task asking them to fill in the definite determiner for each of the 96 items shown in the experiment. Furthermore, in this post-test, they were asked to rate the certainty of their answer on a 3-point scale, as well as indicate whether they thought they had learnt the determiner during the experiment by means of a yes/no response. Subsequently, participants performed the LexTALE test, the score of which gives an estimate of vocabulary size that has been shown to give a good indication of overall language proficiency (Lemhöfer and Broersma, 2012), and completed a motivation and anxiety questionnaire on a computer. The questionnaire included two positively worded and two negatively worded questions on each of four topics (motivation, perfectionism, confidence and perseverance) regarding their learning of Dutch as a second language. The 16 statements were based on the Attitude and Motivation Test Battery (Gardner, 1985) supplemented by some of our own statements (see Appendix A in Supplementary Material).

### ERP data collection

EEG activity was recorded with active electrodes mounted on an elastic cap (ActiCAP, Brain Products GmbH, Gilching, Germany) from 60 scalp sites, based on the extended international 10–20 system (including mastoids and ground). The ground electrode was placed at AF7. The vertical electro oculogram (EOG) was recorded above and below the right eye; horizontal EOG was recorded from electrodes positioned at the outer canthus of the left and right eye. Electrode impedance was kept below 10 kΩ. Potentials were online referenced to an electrode placed on the left mastoid. The EEG and EOG were recorded continuously, and converted with a 16-bit resolution and a 500 Hz sampling rate using two BrainAmp DC amplifiers in combination with BrainVision Recorder software (Brain Products GmbH, Gilching, Germany). Recording filters were set to a low cut-off of 0.016 Hz and a high cut-off of 125 Hz.

EEG data was processed and analyzed using EEGLAB (Delorme and Makeig, 2004), which runs in the MATLAB environment (Mathworks, Natick, MA). Data of all participants were re-referenced offline to a common average based on all electrodes, followed by application of two finite impulse response filters: a high pass filter at 0.1 Hz in combination with a low pass filter at 30 Hz to eliminate slow drifts and high frequency artifacts. Before ICA decomposition, segments were created for the purpose of file size reduction. Data was segmented into long epochs time-locked to stimulus presentation that included both the response and the feedback presentation. A baseline correction was performed on the 200 ms time window prior to stimulus presentation, such that later segmented response- and feedback-locked data were based on the same baseline. Furthermore, large artifacts were rejected using the joint probability tool implemented in EEGlab. This procedure was conducted for the correct and incorrect datasets separately, so as to avoid rejecting error related data as artifacts. Improbable data were defined based on 5 standard deviations from channel means, which deleted on average 2.8 trials (*SD* = 2.4) for the correct and 7.1 (*SD* = 4.0) trials for the incorrect dataset per participant. An independent component analysis (Infomax algorithm) was performed on the segmented data of every subject (correct and incorrect sets combined). Based on 60 scalp electrodes, an equal number of components was computed, which were screened for eye, muscle and heartbeat artifacts by means of visual inspection of the topographic map, power spectrum and activity over trials. Where observed, the respective component was removed before ICA back transformation (on average 11.1 components per participant were rejected, *SD* = 4.0). The resulting epochs were visually inspected for remaining eye artifacts, which were deleted from the dataset (*M*_correct_ = 1.3, *SD* = 1.4; *M*_error_ = 1.1, *SD* = 1.3).

The resulting dataset was re-epoched to form response-locked and feedback-locked segments, both of which had a 200 ms baseline. These segments were averaged for correct and incorrect trials per round per participant, which formed the basis of subsequently created grand averages. We used a minimum of 5 trials per cell as a criterion to include the participant in the analyses. In line with previous studies, the neural response to response-locked (ERN) data were quantified as trough-to-peak amplitudes (Overbeek et al., 2005; Wessel et al., 2011), which is a measure that is independent of baseline correction. To maintain consistency within the study, the feedback-locked (FRN) data were quantified in a similar way (e.g., Cohen et al., 2007; Ferdinand et al., 2012; Glienke et al., 2015). Search windows for the peak and trough latencies were defined based on visual inspection of the grand averages. Within these windows, the difference between minimal (trough) and maximal (peak) voltage levels was calculated.

### Data analysis

To examine a change in the performance and electrophysiological responses to error and correct responses, we were interested in effects of response accuracy over rounds. This was analyzed for a number of dependent variables. To characterize the learners' behavioral performance, we first analyzed their gender decisions in terms of response times (RTs) and accuracy scores, as well as RTs on trials following errors, as these typically show post-error slowing effects, which is considered a sign of behavioral adjustment in the context of error monitoring (Danielmeier and Ullsperger, 2011). To answer our specific research questions on internal error detection and feedback processing, we examined response-locked and feedback-locked waveforms respectively as dependent variables. Unless stated otherwise, behavioral and ERP measures were analyzed as dependent variables in two-factor repeated measures ANOVAs with correctness (either response accuracy or feedback type, two levels) and round (three levels) as factors. In order to examine interaction effects between correctness and round, subsequent paired samples *t*-tests were performed to compare performance between correct and incorrect conditions per round. Greenhouse-Geisser corrections are reported in case the assumption of sphericity was violated. Additionally, we looked whether the feedback-related EEG response in one round was predictive of response accuracy in a subsequent round. Furthermore, correlation analyses were performed to assess relations between different ERP effects as well as behavioral performance and individual difference measures. The Results section only reports findings on target items; filler items (i.e., items that were not cognates with incongruent gender) were not analyzed.

## Results

### Behavioral data

Picture naming responses prior to the gender decision task indicated high noun familiarity for target items (*M* = 95%, *SD* = 5, range 85–100%). We therefore included all target items in the behavioral and ERP analyses. Across all rounds, participants made 36% errors on target items in the gender assignment task (which was significantly higher than 17% errors made on filler trials, *p* < 0.001). To verify that the errors produced in the task were not due to responding too fast, we looked at response times. A repeated measures ANOVA on RTs with accuracy and round as factors revealed a main effect of accuracy [*F*_(1, 22)_ = 6.48, *p* = 0.018, $\eta_{p}^{2}$ = 0.228] in combination with an interaction with round [*F*_(1.57, 34.53)_ = 12.24, *p* < 0.001, $\eta_{p}^{2}$ = 0.358]. Subsequent paired samples *t*-tests showed that response times to error responses and correct responses were comparable in round one (*M*_error_ = 1301, *SE* = 51, *M*_correct_ = 1369, *SE* = 97; *t* < 1) and round two [*M*_error_ = 1349, *SE* = 60; *M*_correct_ = 1282, *SE* = 63; *t*_(22)_ = 1.62, *p* = 0.120], while error responses were significantly slower (*M* = 1531, *SE* = 156) than correct responses (*M* = 1129, *SE* = 83) in round three [*t*_(22)_ = 3.89, *p* < 0.001]. This indicates that errors were caused by unstable representations and uncertainty associated with learning rather than action slips (responding too fast). An additional ANOVA on all RTs (targets and fillers combined) with next round accuracy (2 levels: next round correct, next round incorrect) and round (3 levels) furthermore showed an interaction effect [*F*_(2, 44)_ = 4.98, *p* = 0.011, $\eta_{p}^{2}$ = 0.185]. Subsequent paired samples *t*-tests showed that in round one responses following error trials (*M* = 1333 ms, *SE* = 64) were significantly slower than RTs for items following correct trials [*M* = 1241 ms; *SE* = 51; *t*_(22)_ = 3.81, *p* < 0.001], indicating post-error slowing. Such a post-error slowing effect was not observed for rounds two and three (*t*'s < 1).

**Figure 2:**
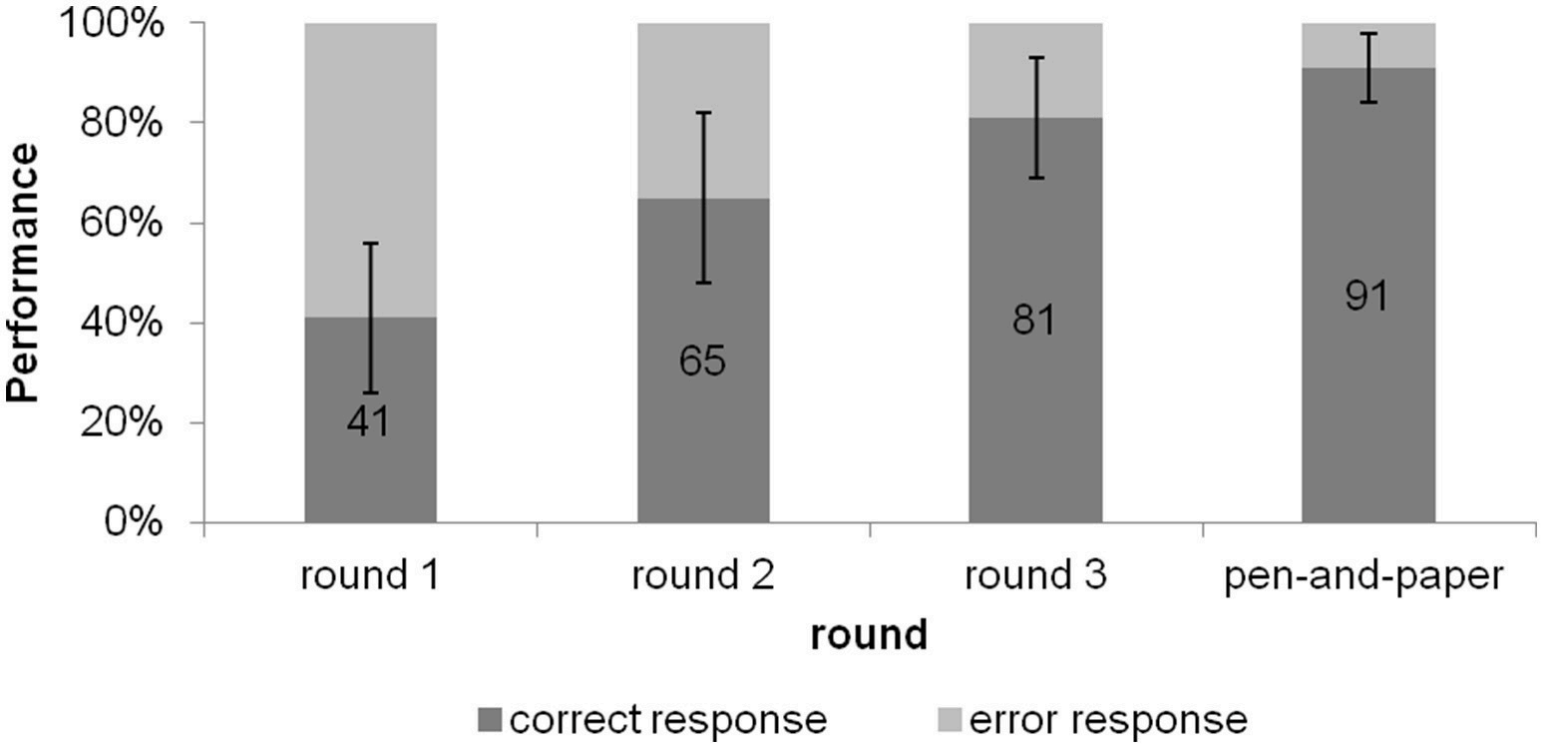
**Mean scores per round (%) for target items on gender assignment task**. Error bars reflect standard deviations for correct responses. Numbers indicate percentage correct. Accuracy scores for pen-and-paper test indicate performance on a written post-test participants took after the EEG experiment.

To analyze performance on target items over rounds, we conducted a one-factor repeated measures ANOVA on accuracy scores with round (4 levels: 3 rounds and post-test) as factor, which showed a main effect of round on accuracy scores, [*F*_(3, 66)_ = 60.21, *p* < 0.001, $\eta_{p}^{2}$ = 0.732]. Planned contrasts (repeated) indicated that accuracy scores significantly improved after every round of feedback during the EEG experiment as well as in the pen-and-paper post-test, assigned 15 min after the EEG experiment (all *p*'s < 0.005) (see Figure 2). As part of this post-test, we also collected certainty ratings for every gender response. A paired samples *t*-test on certainty ratings for targets (rated between 0 for unsure to 2 for sure) indicated higher certainty scores for correct items (*M* = 1.80; *SE* = 0.07) compared to incorrect items (*M* = 1.49; *SE* = 0.04) on the post-test [*t*_(22)_ = 7.03, *p* < 0.001].

### Event-related potentials

**Figure 3:**
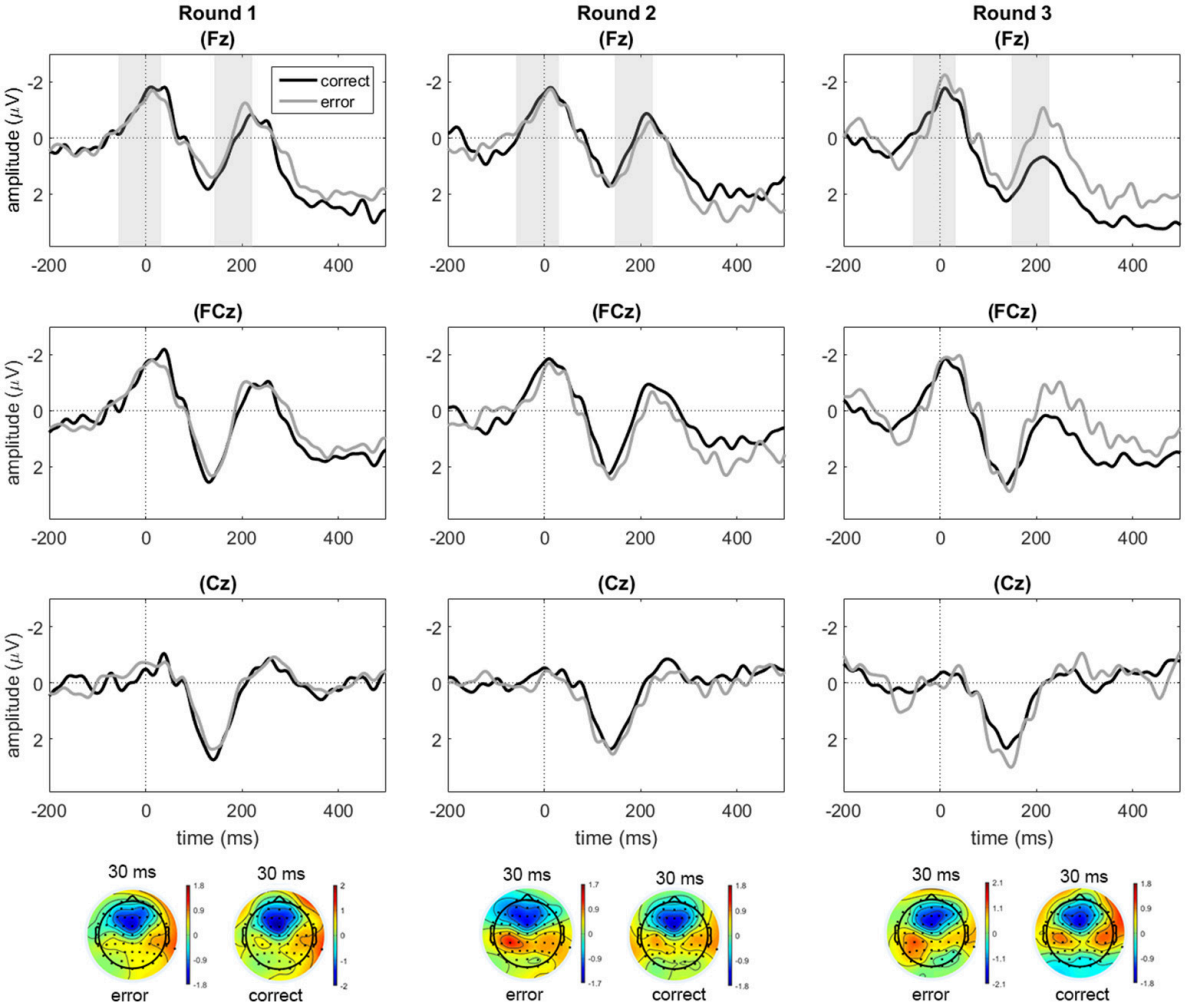
**Response-locked data for correct and error responses in rounds 1, 2, and 3 at electrodes Fz, FCz, and Cz**. Only for the purpose of depicting the data, an additional baseline correction was performed over the pre-response window starting from −200 ms. Shaded areas indicate the average time windows of the trough-to-peak amplitudes from the trough latency to the peak latency.

Waveforms for electrodes Fz, FCz, and Cz were inspected for the occurrence of frontally distributed response-locked negativities. As shown in Figure 3, such negativities were present in the response-locked data at Fz and FCz, but not for Cz. Initial analyses on Fz and FCz showed no qualitative differences regarding the effects of accuracy and round between the two electrodes. Reported analyses for ERN are based on electrode Fz, because amplitude differences between conditions were maximal here. Similarly, FRN analyses were conducted on electrode Fz to maintain consistency within the study. Because peak latencies vary across participants and are therefore not always clearly visible in the grand average waveform, mean values and peak latencies for the trough-to-peak measures are also given separately (see Tables 2, 3). In addition to the ERN and FRN measures, we measured subsequent components. The response-locked data revealed a second negative peak after the ERN/CRN, which was captured by another trough-to-peak measure. The feedback-locked data P300 effect was captured by a mean amplitude over electrode clusters.

**Table 2:** **Average trough-to-peak amplitude measures in μV (*SD*) at Fz for response-locked data per round**.

**Round**	**Amplitude error-related negativity**			**Amplitude second negativity**		
	**Error (ERN)**	**Correct (CRN)**	**Difference**	**Error amplitude**	**Correct amplitude**	**Difference**
1	3.76 (1.46)	4.34 (1.91)	−0.58	3.87 (1.98)	3.99 (1.96)	−0.12
2	4.18 (2.12)	3.82 (1.91)	0.36	4.31 (1.87)	3.47 (1.53)	0.84
3	6.37 (4.79)	3.44 (1.81)	2.93	5.47 (4.25)	2.89 (1.55)	2.58

**Table 3:** **Average trough-to-peak amplitude in μV (*SD*) at Fz for feedback-locked data per round**.

**Round**	**Feedback related negativity**		**Difference**
	**Negative feedback amplitude**	**Positive feedback amplitude**	
1	5.54 (2.40)	4.63 (1.93)	0.91
2	6.55 (2.25)	4.63 (1.98)	1.92
3	8.13 (3.79)	3.48 (2.29)	4.65

#### Response-locked data

We compared the trough-to-peak amplitudes for correct and erroneous responses across rounds to test if a response-related negativity for errors would reveal signs of internal monitoring of gender responses. In line with previous studies (Danielmeier et al., 2009), the time window of the negative peak for ERN and CRN measures was determined to be ranging from response onset to 150 ms after the response (*M*_error_ = 29 ms; *SD* = 25; *M*_correct_ = 34 ms; *SD* = 32), while the preceding positive peak (trough) was measured between 100 ms before response onset and the negative peak (*M*_error_ = −61 ms; *SD* = 41; *M*_correct_ = −63 ms; *SD* = 42).

A repeated measures ANOVA on the trough-to-peak amplitudes for Fz showed trends toward main effects of response accuracy [*F*_(1, 22)_ = 4.26, *p* = 0.051, $\eta_{p}^{2}$ = 0.162], and round [*F*_(2, 44)_ = 2.62, *p* = 0.084, $\eta_{p}^{2}$ = 0.107], and a significant interaction between response accuracy and round [*F*_(1.50, 32.88)_ = 10.16, *p* = 0.001, $\eta_{p}^{2}$ = 0.316]. Subsequent paired samples *t*-tests for each round indicated marginally larger trough-to-peak amplitude differences for correct compared to erroneous responses in round one, thus reflecting a difference in the opposite direction [*t*_(22)_ = −1.93, *p* = 0.067], no difference between error and correct responses in round two (*t* < 1), and significantly larger amplitude differences for errors compared to correct responses in round three [*t*_(22)_ = 3.16, *p* = 0.005], representing a small ERN effect in the expected direction (see Table 2 for values, and Figure 3).

Apart from the early peak, the response-locked data also showed a second negative peak around 200 ms post-response (see second shaded area in Figure 3). To analyze this component, we computed a trough-to-peak difference for Fz based on the negative peak amplitude in the 200–300 ms post-response time window, which was compared to the preceding positive peak within 100 ms prior to the peak. A repeated measures ANOVA on the amplitudes of this second negativity showed a main effect of accuracy [*F*_(1, 22)_ = 8.26, *p* = 0.009, $\eta_{p}^{2}$ = 0.273], in combination with a significant two-way interaction between accuracy and round [*F*_(1.39, 30.58)_ = 4.63, *p* = 0.028, $\eta_{p}^{2}$ = 0.174]. There was no main effect of round (*F* < 1). Follow-up comparisons on the effect of accuracy per round indicated no significant difference with regard to the second negativity between errors and correct responses in round one (*t* < 1), but a significantly larger trough-to-peak difference for errors compared to correct responses in round two [*t*_(22)_ = −2.24, *p* = 0.035], where the effect is numerically small (see Table 2) and in round three [*t*_(22)_ = −2.79, *p* = 0.011], where the effect is clearly visually present. The second negative peak thus seemed to roughly resemble the pattern observed for the ERN component.

In sum, the response-locked data pointed to an increasing ERN effect that developed in the course of the experiment, moving from a trend toward larger CRN than ERN amplitudes in round one to an ERN that was significantly larger than the CRN in round three. This effect co-occurred with a second negativity that showed a similarly increasing difference between correct and error responses.

#### Feedback-locked data

To examine neural responses to positive and negative feedback across rounds, we first of all looked at the FRN. In both conditions, the FRN was calculated as the amplitude difference between the negative peak within the time-window of 200–380 ms post-feedback (negative feedback: *M* = 307, *SD* = 49; positive feedback: *M* = 300; *SD* = 49) and the preceding positive peak up to 100 ms before the negative peak (negative feedback: *M* = 238, *SD* = 55; positive feedback: *M* = 232; *SD* = 49), making the time window of the preceding peak dependent on the later peak latency (see Table 3 for amplitudes).

**Figure 4:**
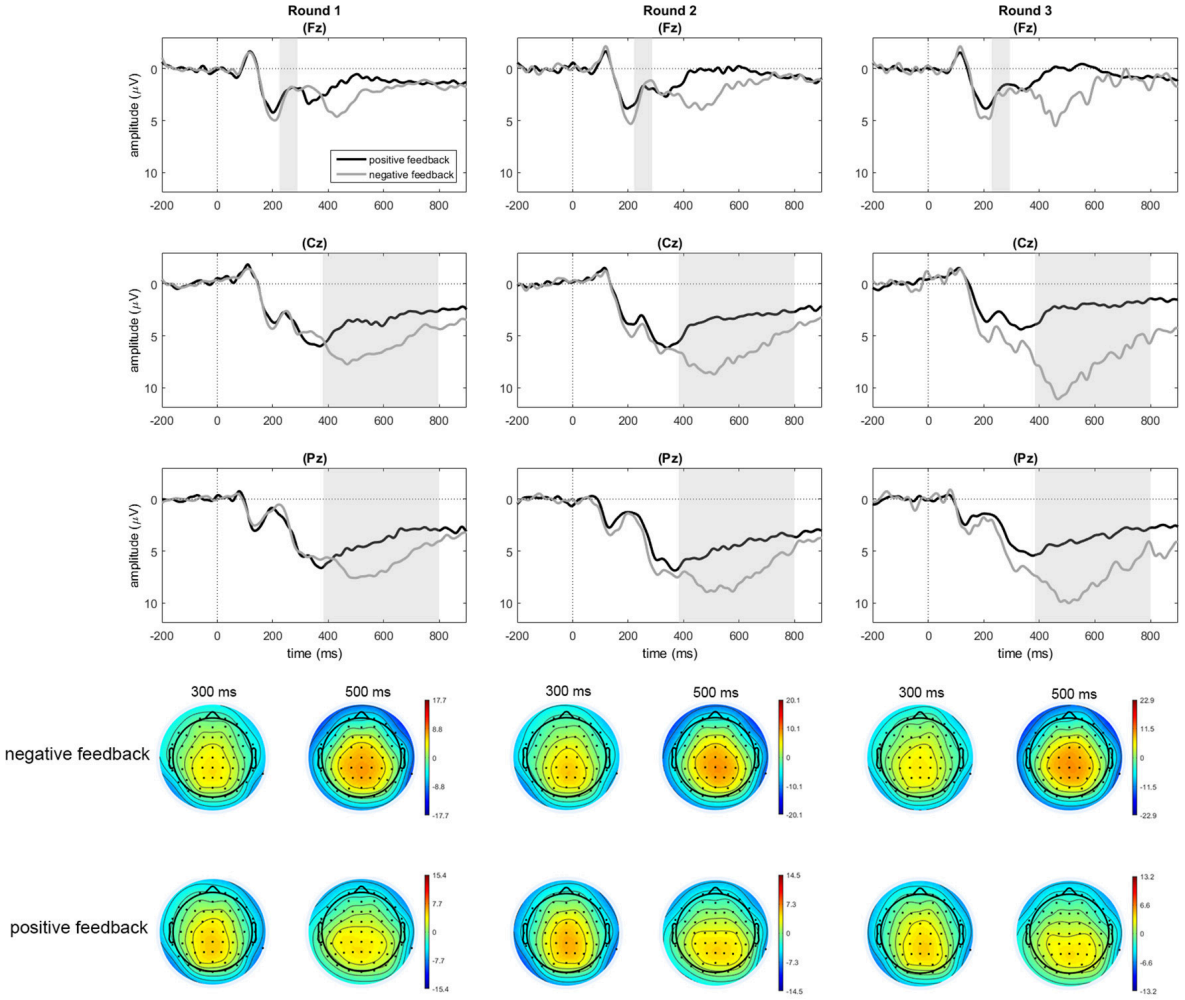
**Feedback-locked data for positive and negative feedback in rounds 1, 2, and 3 at electrodes Fz, Cz, and Pz**. Only for the purpose of depicting the data, an additional baseline correction was performed over the pre-feedback window starting from −200 ms. Shaded areas indicate the average time windows of the FRN trough-to-peak measure (Fz), running from the trough latency to the peak latency, and the time window of the mean amplitude for the P300 measure (Cz, Pz).

A repeated measures ANOVA on the trough-to-peak measure at Fz with feedback type and round as factors indicated a main effect of feedback type [*F*_(1, 22)_ = 46.11, *p* < 0.001, $\eta_{p}^{2}$ = 0.677] and an interaction between feedback type and round [*F*_(2, 44)_ = 12.70, *p* < 0.001, $\eta_{p}^{2}$ = 0.336]. There was no main effect of round, [*F*_(2, 44)_ = 1.47, *p* = 0.242, $\eta_{p}^{2}$ = 0.062]. Paired samples *t*-tests to examine the effect of feedback for every round indicated that negative feedback yielded significantly larger negative trough-to-peak amplitudes than positive feedback in round one [*t*_(22)_ = 2.14, *p* = 0.043], round two [*t*_(22)_ = 3.67, *p* = 0.001], and round three [*t*_(22)_ = 6.32, *p* < 0.001]. This numerically large FRN effect in round three (see Table 3) is not visible in the depicted grand averages (Figure 4) due to smearing of the effect caused by variation in peak latencies among participants. Note that instances of negative feedback constituted only 19% of responses in round three, making the measure noisier than in preceding rounds.

To account for a possible noise bias in the trough-to-peak measure, we also quantified the FRN component as a mean amplitude, which centered 40 ms around the latency of the negative peak between 200 and 380 ms. A two-factor repeated measures analysis on the mean amplitudes indicated no significant main effect of feedback type [*F*_(1, 22)_ = 2.08, *p* = 0.163, $\eta_{p}^{2}$ = 0.086], nor an interaction between feedback type and round (*F* < 1). The data did show a numeric difference between negative and positive feedback in the same direction as the reported trough-to-peak measure, with more negative amplitudes for negative feedback compared to positive feedback across all three rounds.

The feedback-locked data showed that the FRN effect was followed by a broad ranging P300 effect. As is common in the literature, we analyzed this effect by looking at the mean amplitude in the time window between 380 and 800 ms after feedback onset, based on visual inspection of the grand average. To compare P300 components for positive and negative feedback, we performed analyses on three electrode clusters along the midline (based on methodology described in Martin et al., 2010) for which averages were computed over 5 electrodes each: fronto-central (F1, F2, FC1, FCz, FC2), parieto-central (C1, Cz, C2, CP1, CP2), and parieto-occipital (CPz, P1, Pz, P2, POz). A repeated measures ANOVA was performed on the mean amplitudes per cluster with feedback type (2 levels), round (3 levels), and region (3 levels) as factors. The analyses yielded a significant main effect of feedback type [*F*_(1, 22)_ = 43.65, *p* < 0.001, $\eta_{p}^{2}$ = 0.665], as well as a trend toward an effect of round [*F*_(2, 44)_ = 2.99, *p* = 0.061, $\eta_{p}^{2}$ = 0.120] in combination with a significant two-way interaction between feedback type and round [*F*_(1.51, 33.12)_ = 8.68, *p* = 0.002, $\eta_{p}^{2}$ = 0.238]. Interactions with region were not significant (*p* > 0.20). To follow up on the feedback type by round interaction, repeated measures ANOVAs were performed on the amplitudes per round (across the three regions) to test for the effect of feedback type in each round. These analyses indicated main effects of feedback type for round one [*F*_(2, 21)_ = 17.12, *p* < 0.001, $\eta_{p}^{2}$ = 0.438], round two [*F*_(2, 21)_ = 35.08, *p* < 0.001, $\eta_{p}^{2}$ = 0.615], and round three [*F*_(2, 21)_ = 41.45, *p* < 0.001, $\eta_{p}^{2}$ = 0.653]. In all cases, mean amplitudes for negative feedback were larger than those for positive feedback. To characterize the interaction, we also looked at the effect of round in repeated measures ANOVAs for positive and negative feedback separately, which indicated no effect of round in the amplitudes for positive feedback [*F*_(2, 44)_ = 1.23, *p* = 0.302, $\eta_{p}^{2}$ = 0.053], while an effect of round was present in the amplitudes for negative feedback [*F*_(1.47, 32.36)_ = 6.80, *p* = 0.007, $\eta_{p}^{2}$ = 0.236]. Repeated contrasts for the factor round indicated a significant increase in amplitudes for negative feedback between rounds one and two (*p* = 0.004), while amplitudes in rounds two and three were not different (*p* = 0.183).

In sum, the feedback-locked data showed larger FRN and P300 deflections following negative compared to positive feedback across all three rounds; both effects increased in size in the course of the experiment.

#### Signs of behavioral learning in the feedback-locked data

To examine if the processing of negative feedback in one round was indicative of performance in the subsequent round, we split the neural responses for negative feedback into responses that led to learning and those that remained instable. We therefore divided the negative feedback responses of round one (59% of responses) into responses that were subsequently correct in rounds two and three (29% of responses) and those that were incorrect in round two (23% of responses), which may or may not have been corrected in round three. Similar analyses for the negative feedback of round two according to behavioral accuracy in round three were not feasible because of too few data points for not-learned items in that round.

**Figure 5:**
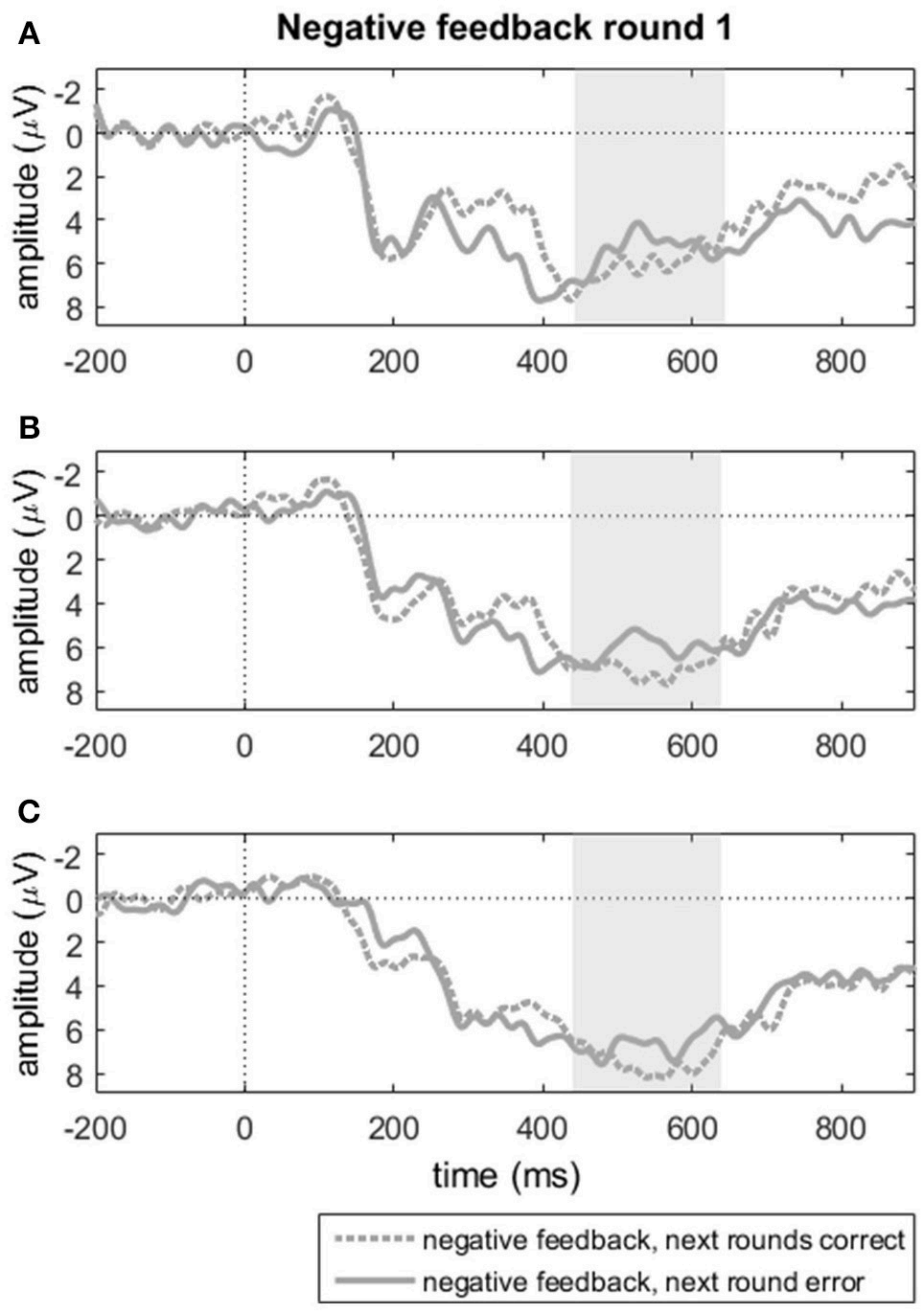
**Negative feedback in round one split by behavioral accuracy in subsequent rounds for (A)** FCz, **(B)** Cz, and **(C)** CPz. “Next rounds correct” indicates stable correct responses in rounds 2 and 3; “next round error” reflects incorrect responses in round 2, that could be either correct or incorrect in round 3. Shaded areas indicate the time window of the P300 mean amplitude measure.

A paired samples *t*-tests on the FRN trough-to-peak measures for negative feedback in round one on electrode Fz showed no difference between subsequently learned and not-learned items (*t* < 1). Alternatively, we also computed mean amplitudes over the 6 midline electrodes between 250 and 450 ms, where the grand average suggested a difference between subsequently correct and incorrect responses. A repeated measures analysis with subsequent response accuracy (2 levels: correct, incorrect) and electrodes (6 levels: Fz, FCz, Cz, CPz, Pz, POz), however, indicated no significant effects of response accuracy, nor an interaction between accuracy and electrode (*F*'s < 1). Similarly, we compared the mean amplitudes for the P300 component for the same groups of incorrect trials in round one in terms of subsequent accuracy. Visual inspection of the ERP grand averages indicated that this component diverged for learnt vs. unlearnt items only in a much smaller window than the one used for the P300 reported above; we tentatively tested whether this divergence was significant. Mean amplitudes were computed over the time window from 450 to 650 ms post-feedback (see Figures 5A–C). We analyzed the effects of accuracy in the next round (2 levels) and region (3 levels) on the three midline clusters reported above. A repeated measures ANOVA with these two factors showed a trend toward a main effect of accuracy in the next round [*F*_(1, 22)_ = 3.19, *p* = 0.088, $\eta_{p}^{2}$ = 0.127] with larger P300 amplitudes for negative feedback items that were answered correctly in the following round (*M* = 5.52, *SD* = 3.75) compared to when errors were repeated (*M* = 4.31, *SD* = 3.37). The two-way interaction among subsequent accuracy and region was not significant, [*F*_(2, 44)_ = 2.20, *p* = 0.123, $\eta_{p}^{2}$ = 0.091]. This pattern of results points to some indication of a P300 amplitude during feedback processing that was predictive of learning, though in a very restricted time window.

### Correlations among different ERP measures

We checked for correlations among the sizes of the different ERP effects to see if patterns among the different measures of performance monitoring were related. To test for relations between the two response-locked measures, we correlated the ERN effect and the second negativity in each round (see Table 4). Bonferroni-corrected correlations (0.05/3 = 0.018) across participant means between the ERN and second negative peak (both computed as the difference between error and correct conditions) for each round showed significant positive correlations in round one [*r*_(23)_ = 0.53, *p* = 0.010] and round three [*r*_(23)_ = 0.53, *p* = 0.009], which suggested that the occurrences of the ERN and the second negativity were related. Perhaps due to the absence of a difference between ERN and CRN measures in round two, there was no such relationship between the two measures in this round [*r*_(23)_ = −0.02, *p* = 0.927].

**Table 4:** **Correlation matrix of response-locked an feedback-locked ERP measures per round**.

	**Second negativity effect**			**FRN effect**			**Parieto-occipital P3 effect**		
	**R1**	**R2**	**R3**	**R1**	**R2**	**R3**	**R1**	**R2**	**R3**
ERN effect R1	0.53^**^	0.46^*^	0.06	0.07	0.56^**^	0.20	0.26	0.22	0.25
ERN effect R2	0.03	−0.02	−0.10	0.03	0.26	−0.02	0.13	0.17	0.15
ERN effect R3	−0.04	0.30	0.53^**^	0.04	0.27	0.61^***^	0.52^*^	0.52^*^	0.70^***^

Significance level:

*** <0.006; levels of

** *< 0.01*,

* *<0.05 were not significant with the Bonferroni correction, N = 23. ERN, second negativity and FRN effects reflect difference measures based on electrode Fz*.

To test if feedback-locked responses in preceding rounds could predict the occurrence of an ERN effect in the final round, we computed correlations between the ERN effect and FRN and P300 effects (see Table 4). Bonferroni corrections were applied per dependent variable, based on three measures for the FRN and P300 effects that were correlated with the three rounds of ERN effects (significance level = 0.05/(3^*^3) = 0.006). There were no significant correlations between FRN effects (measured as the difference in trough-to-peak amplitudes between negative and positive feedback per round) and ERN effects (measured as the difference in trough-to-peak amplitudes between incorrect and correct responses per round), apart from a correlation between the size of the ERN and FRN effects in round 3 [*r*_(23)_ = 0.61, *p* = 0.002; see Table 4]. ERN effects in round three furthermore correlated with P300 effects (measured as the difference in mean amplitudes between negative and positive feedback at the parieto-occipital electrode cluster per round) in round three [*r*_(23)_ = 0.70, *p* < 0.001], and showed correlations with P300 effects in rounds one and two that missed significance when the Bonferroni correction was applied [*r*_(23)_ = 0.52, *p* = 0.011, in both rounds]. The positive correlations thus suggested that larger FRN and P300 effects in learners were associated with larger ERN effects in the third round (see Table 4).

### Individual differences

To examine individual differences in behavioral and neural responses, the RT, accuracy, and EEG data were correlated with measures of six different individual characteristics, including Dutch vocabulary size (LexTALE), average self-rated certainty for items on the task (assessed after the EEG experiment), and self-rated language learning motivation (general motivation, perfectionism, perseverance, and confidence ratings; see Table 5 for all correlations). To correct for multiple comparisons in the correlation matrix, we applied a Bonferroni correction per dependent variable, based on six measures for three rounds of RT, accuracy, ERN and FRN effects (significance level = 0.05/(6^*^3) = 0.003). In terms of behavioral measures, participants' scores on the LexTALE test showed a positive correlation with accuracy scores in round one, which decreased in the remainder of the experiment, and trends toward negative correlations with RTs in rounds two and three for correct responses. Together these correlations are a sign that participants with larger Dutch vocabulary size performed the gender assignment task faster (on correct responses) and more accurately (see Table 5). Mean certainty ratings that were obtained with the gender responses in the post-test were positively correlated with accuracy on this pen-and-paper post-test [*r*_(23)_ = 0.62, *p* < 0.01], but also showed an increasing correlation with behavioral accuracy in the three preceding rounds of the online gender assignment task (see Table 5), which implied that participants indicated to be more certain when their previous responses on the task were more often correct. In general, motivation scores did not predict performance on the decision task concerning our target nouns (incongruent cognates). Yet, correlations were observed between accuracy in round two and perfectionism scores, as well as trends toward negative correlations between RTs for correct and error responses in rounds one and two and mean confidence ratings. This points to higher levels of accuracy for more perfectionist learners and faster responses for more confident learners.

**Table 5:** **Correlation matrix of proficiency measure and self-ratings on certainty and motivation with behavioral and ERP measures per round**.

	**RT correct**			**Accuracy**			**ERN effect**			**FRN effect**		
	**R1**	**R2**	**R3**	**R1**	**R2**	**R3**	**R1**	**R2**	**R3**	**R1**	**R2**	**R3**
LexTALE	−0.39	−0.55^**^	−0.47^*^	0.62^***^	0.47^*^	0.41	0.44^*^	−0.03	0.05	0.01	0.53^**^	0.03
Certainty rating	−0.29	−0.29	−0.36	0.51^*^	0.61^***^	0.75^***^	0.47^*^	0.15	0.65^***^	−0.02	0.49^*^	0.62^***^
Motivation	0.14	−0.01	−0.10	0.13	0.31	−0.08	0.19	−0.14	−0.31	−0.12	0.17	0.22
Perfectionism	−0.08	−0.21	−0.12	0.37	0.62^***^	0.40	0.21	0.26	0.31	−0.20	0.32	0.48^*^
Confidence	−0.44^*^	−0.48^*^	−0.34	0.21	0.06	−0.12	0.29	0.20	−0.03	−0.02	0.37	−0.19
Perseverance	−0.16	−0.30	−0.07	0.52^*^	0.51^*^	0.10	0.53^**^	−0.01	0.01	−0.38	0.57^**^	0.00

Significance level:

*** <0.003; levels of

** *< 0.01*,

* *<0.05 were not significant with the Bonferroni correction, N = 23*. *Correlations are based on Spearman's rho values; for LexTALE score Pearson's r is reported. ERN effect reflects the amplitude difference ERN-CRN; the FRN effect is based on the amplitude difference FRN negative-FRN positive*.

With respect to neural responses, the six individual difference measures were correlated with ERN and FRN effects, calculated as the differences in amplitude between error and correct responses and negative and positive feedback respectively. The size of the ERN effect only showed a significant positive correlation with certainty ratings in round three, which meant that more certainty about responses at the pen-and-paper test was preceded by a larger ERN for errors compared to correct responses in the third round. Furthermore, there was a trend between the size of the ERN effect in round one and perseverance ratings, which suggested that more perseverant learners displayed larger CRN than ERN components, as the effect was in the opposite direction in this round. The size of the FRN effect in round three was also shown to be correlated with certainty ratings, indicating that more certain learners displayed larger amplitude differences between negativities for positive and negative feedback. Furthermore, the FRN data point to trends toward correlations in round two that suggest larger FRN effects for more proficient and more perseverant learners. The increased neural response of the most proficient and motivated learners after two rounds of feedback seems to level out in the remainder of the experiment.

In sum, behavioral performance on the gender assignment task was shown to be related to vocabulary size and certainty ratings. ERN and FRN effects were also shown to be larger for learners who had indicated to be more certain.

## Discussion

The present study examined relatively proficient L2 learner's performance on a gender assignment task involving corrective feedback to investigate how L2 learners acquire stable gender representations. We aimed to find out whether the learners showed signs of internal error monitoring, as an indication of learning, before and after receiving feedback and examined how they responded to feedback. The behavioral results indicated that the L2 learners made errors that were of the representational kind (due to a lack of knowledge) rather than action execution errors (pressing the wrong button), as revealed by slower RTs for errors compared to correct responses (see Wickens, 1992). On average, learners improved from accuracy rates of 41% before the training phase to 91% in a final pen-and-paper test after the third and final round of feedback. Attentional processes related to learning were furthermore reflected by post-error slowing in the first round. Together, this provides first evidence for initially weak L2 representations of grammatical gender, followed by behavioral learning in the context of a relatively short training session for a feature that, as our and previous studies show, is difficult to acquire by this population (see also, e.g., Lemhöfer et al., 2010).

To examine the external and internal monitoring processes involved during initial processing and learning, we investigated the occurrence of response-locked and feedback-locked effects. In what follows, we will discuss performance on the task with regard to internal error detection and feedback processing as neural signatures in relation to learning.

### Detection of errors

To find out if the quite proficient L2 learners, who were not entirely naive concerning gender representations in their L2, showed signs of internal error detection in a learning task, we investigated the occurrence of the ERN. In all three rounds, we observed an early negative peak that closely followed the moment at which an erroneous response was made, which we interpreted as an ERN. Additionally, we observed a similar sized CRN for correct responses. Along with behavioral improvement, the neural results indicated a change in response-locked negativities over rounds. For responses in the first round, before any feedback was given, the CRN was slightly larger than the ERN. Following one round of feedback on performance accuracy and presentation of correct responses, the results of round two showed increased behavioral performance, while no difference was present between ERN and CRN. After participants had been presented with corrective feedback twice, further improved behavioral performance was accompanied by a small but significant ERN effect (the difference between ERN and CRN) in trough-to-peak measures for remaining errors in round three. The results of round one thus suggested a reverse ERN effect, while round two indicated no difference between the ERN and CRN, and round three pointed to an ERN effect in the expected direction.

The observed pattern of a negative peak for both errors and correct responses, which appears relatively early, does not resemble a typical performance monitoring ERN effect with an almost flat waveform for correct responses (cf. Gehring et al., 2011). This fits with the absence of action execution errors as indicated by the RT data. The similar-sized ERPs do resemble previous findings on decisions under uncertainty, for example, in memory retrieval tasks following a learning phase. Rodriguez-Fornells et al. (2004) showed that learners who had been presented with competing response alternatives alongside targets during the learning phase (errorful learning), showed similar sized ERN and CRN components for both correctly recognized target items (hits) and incorrectly recognized competing response alternatives (false alarms) in the retrieval phase. Learners who had only been presented with the correct target response during learning (errorless learning) on the other hand, showed a much larger ERN component for false alarms than for hits. This suggests that activation of competing responses leads to conflict during memory retrieval, such that errors are less easily detected (see also Heldmann et al., 2008a).

Similarly, the L2 learners in the present study, who performed a learning task involving memory encoding, may have experienced interference from competing L1 representations stored in memory. Poor behavioral performance in the first round suggested that learners were influenced by their L1 representations. The gender representations for all cognate targets were incongruent between Dutch and German, which is known to give rise to incorrect L1 transfer during L2 acquisition (Lemhöfer et al., 2008, 2010). Knowledge of the L1 can thus be a reason for incomplete or incorrect L2 representations. This may also explain the opposite pattern of results in round one, before feedback: If learners responded correctly to incongruent cognates, they could have “felt” they were making an error (in terms of their L1), because they also activated their L1 representations. This could have given rise to large a CRN, which was actually larger than the ERN in this round. This interpretation would fit with the previous finding that the P600 response to gender agreement violations in German learners of Dutch is based on subjective, sometimes incorrect and L1-based representations rather than on objective correctness (Lemhöfer et al., 2014). Negative L1 transfer may thus first of all have caused errors and incorrect intuitions about L2 gender in initial stages of the experiment, which were presumably destabilized by the first feedback. These recently destabilized representations were probably not yet strong enough to elicit differences between observed ERN and CRN components in round two (after the first feedback). However, the non-significant difference between ERN and CRN waveforms in round two could also suggest different effects (presence of ERN for some items, and reverse ERN for others) canceled each other out. L1 interference could thus be one reason for difficulty during error detection, but the large CRN could additionally be explained in terms of response uncertainty (i.e., weak memory traces of the correct gender-noun association) in relation to incomplete learning.

The relation between uncertainty and the occurrence of a large CRN component has previously been pointed out in studies that manipulated perceptual uncertainty (Pailing and Segalowitz, 2004; Navarro-Cebrian et al., 2013; see also Coles et al., 2001). These studies showed that uncertainty about performance accuracy gives rise to similar negativities for error and correct responses, because errors cannot be judged properly, yielding smaller ERNs for undetected errors while correct responses are deemed incorrect, leading to CRNs. A similar account may hold for the uncertainty present in the learning situation we tested, albeit of a different nature, due to memory rather than perceptual failure. In the present study, the similar magnitudes of response-related negativity for errors and correct responses imply that the ERN effect (the difference in amplitudes) when considered a reflection of a prediction error (as hypothesized by Holroyd and Coles, 2002) on performance accuracy is relatively small. This would agree with current ideas on the predictive brain reflected in the Predictive Processing account (Feldman and Friston, 2010; Friston, 2010; Kwisthout et al., 2016), which hypothesizes that when certainty regarding outcomes is low, the brain's predictions on correct performance are not very precise, and consequently the prediction error as a reflection of surprise to the brain's prediction on response outcome (i.e., the ERN effect) can only be small. Further evidence for the role of uncertainty was found in correlation effects regarding certainty ratings obtained at the end of the learning session, which showed that the most important individual difference factor influencing the observed effects was certainty. More certainty about acquisition of the correct determiner was related to higher behavioral accuracy and larger ERN effects. The present results thus seem to indicate that acquisition of features of an L2 that are similar, but not the same as the L1, yields much uncertainty due to unstable representations or difficulty in error detection due to competing representations, which may likewise result in uncertainty.

Although the difference between response-locked negativities for correct and incorrect responses remains small throughout the experiment, a small ERN effect (i.e., a larger ERN than CRN) could be observed in round three. In the present study, this may be considered the first signature of internal error detection based on stable and correct gender representations as a result of training. Such evidence for error detection directly after learning replicates the small ERN effect for relatively low proficient Dutch learners of German observed by Davidson and Indefrey (2011).

Across rounds, the ERN and CRN components occurred in combination with a second negative peak. This effect has also been observed in several language studies on error monitoring (Rodriguez-Fornells et al., 2004; Sebastian-Gallés et al., 2006; Davidson and Indefrey, 2011), yet has rarely been accounted for. One paper that does address this finding (Riès et al., 2011) links it to error correction, as discussed by Fiehler et al. (2005). The latter study reports a large second negativity for corrected vs. uncorrected errors. Yet, our design, in which overt error correction was not possible, does not allow for any conclusions regarding error correction. Instead, the finding may be very tentatively related to error awareness that is implied in error correction, as argued below. We observed (trends toward) positive correlations between the difference measures (between correct and incorrect responses) of the second negativity and the ERN across participant means, suggesting the signals co-occurred in the same participants. Given that the second negativity seems predominantly present in paradigms involving language and memory, we assume that the data pattern is characteristic of the response-locked waveforms and may be related to retrieval processes relevant to such tasks. In the present study, the second negative peak could be a sign of on-going evaluation of response options (possibly implying error awareness) for a response that was based on slower memory retrieval in relation to learning. Such an explanation can be related to conceptual proposals on L2 monitoring. L2 production, similar to models of L1 production (Levelt, 1983; Roelofs, 1997; Hartsuiker and Kolk, 2001), is assumed to be subject to internal monitoring to detect and correct errors before, during or after making the error, with the difference that monitoring in L2 is slower and more dependent on attentional control, because L2 processing is less automatized (Kormos, 1999). This reduced automaticity for a newly learnt feature in L2 could result in later awareness of the error (perhaps the actual error detection) that can explain the presence of a second negativity. However, the present findings do not immediately clarify the functional significance of this finding, and future studies should address this issue in more detail.

### Feedback processing

Apart from showing behavioral improvement, the learners in our study were shown to be sensitive to external feedback, evidenced by FRN (trough-to-peak) and P300 effects for negative feedback throughout learning, which indicated that negative feedback led to an evaluation of response accuracy that triggers learning (signaled by FRN) and enhanced memory and attentional processing (signaled by the P300; Ernst and Steinhauser, 2012). Other than expected, both effects did not decrease in the course of learning. The observed FRN effects, signified by larger trough-to-peak differences for negative compared to positive feedback, were statistically comparable for the first two rounds of feedback, and showed an increase in size in the third round. Similarly, an increase in the size of the P300 effect was observed between rounds one and two.

Although a decrease in the FRN, as a result of reduced surprise to feedback on accuracy, was expected, the observation of a sustained FRN effect during learning is not uncommon and can be considered an indication that feedback remains important to learners (Heldmann et al., 2008b). This finding is often reported in gambling tasks when participants are uncertain about response accuracy (e.g., Holroyd et al., 2006; see Heldmann et al., 2008b, for more references). Likewise, feedback in a categorical learning paradigm can remain useful as long as stable representations have not been formed. Other than in a learning paradigm that involves (grammatical) rules (e.g., Davidson and Indefrey, 2011), learners in the present experiment were not able to extract a general rule for the feature. Instead, they had to make an association between the determiner and noun for every word individually, because grammatical gender membership in Dutch is not transparent or bound to general rules. The information to be acquired thus relied on memory processes, which yielded uncertainty, given that many different items had to be remembered at once and incorrectly transferred representations had to be overwritten. This implies that every instance of negative feedback could remain informative if a previous instance of feedback had not been remembered, which can explain why the FRN did not decrease.

As can be observed in the feedback-locked waveforms, the FRN effect is in part due to a difference in the trough (preceding positivity) rather than the subsequent negative peak (see Figure 4), which has previously been pointed out to be a problem for the measurement of this component (San Martín, 2012). In fact, an additional analysis based on mean amplitudes centered around the negative peak yielded a different pattern of statistical results. Although negative feedback gave rise to numerically more negative values in mean amplitudes than positive feedback, this difference was not significant across any of the rounds. A number of other studies investigating the FRN also indicate that it is difficult to capture the effect, in the sense that different measures of the FRN on one and the same dataset sometimes yield different outcomes (e.g., Bellebaum et al., 2010). Moreover, the finding of a difference caused by the preceding positivity rather than the negative peak is not necessarily cause for concern. Because the FRN component is embedded in between a strong visual N1 preceding it, and a strong P300 effect following it, it is not unlikely that either of these surrounding components affect the waveform of the FRN. Given that our visual displays for positive and negative feedback were carefully controlled such that they were as similar as possible between the two conditions, we deem it unlikely that a visual difference in the feedback screens (i.e., the N1 component preceding the FRN) is responsible for the amplitude of the trough around 200 ms. This would imply that the observed difference at the trough might be affected by the subsequent P300 effect. It has often been shown that the large P300 effect superimposed on the FRN can influence the size of the effect and shift it to more positive values (San Martín, 2012, p. 11). We therefore argue that if the large P300 effect for negative feedback indeed influenced the voltage of the FRN, it still indicates a neural response toward negative feedback that can be seen as clear evidence for feedback-guided learning.

The observed increase in the P300 may well reflect increased memory or attentional processing in an attempt to improve performance after one or more instances of negative feedback for a particular word. Another possible explanation concerns probability of negative feedback: When learning takes place and negative feedback occurs more infrequently, the size of neural responses to feedback can increase as their magnitude is inversely related to the probability of occurrence (Polich, 2007; San Martín, 2012).

### The relation between feedback processing and internal error detection

Given the steady increase in behavioral performance in response to feedback, we also examined if it was possible to predict learning (a change from error to correct response on an item) based on how feedback was processed in the previous round (Cohen and Ranganath, 2007; Ernst and Steinhauser, 2012; San Martín, 2012). The present results did not unambiguously show a reflection of on-going learning (behavioral adaptation) in the neural correlates of feedback processing. By comparing the waveforms for negative feedback for words that were correct vs. incorrect in the subsequent round, no differences were seen for the FRN, which is in agreement with some earlier studies that observed an effect of subsequent error correction in P300 window instead (e.g., Butterfield and Mangels, 2003; Ernst and Steinhauser, 2012). Similarly, in our data, a small difference between incorrect responses that were subsequently correct vs. subsequently incorrect items seemed present in a reduced and relatively early time window (450–650 ms) for the P300, but did not reach significance. Additionally, correlation analyses between P300 effects in the first two rounds and the ERN effect in round 3 indicated trends toward a positive relation [*r*_(23)_ = 0.52, *p* = 0.011; Bonferroni corrected] between the feedback-locked and response-locked data. Although neither of these effects were significant, the observed trend in the data could very tentatively be related to attentional orienting or updating (Butterfield and Mangels, 2003; Polich, 2007). Butterfield and Mangels (2003) observed an early frontal feedback-locked P3a (or novelty P3) effect for incorrect responses that were answered correctly during subsequent re-testing, suggesting that this component can predict behavioral adjustment (see also Arbel and Wu, 2016, for recent findings). Fitting with Ernst and Steinhauser's notion (2012) that P300 reflects memory and attentional processing while FRN is an indication of feedback valence, this finding could indicate that the former rather than the latter is indicative of subsequent learning. In case of a larger P300, when more attention was being paid to negative feedback and the simultaneously presented correct response, learners were more likely to remember the correct determiner-noun combination and adapt their behavior in the subsequent round. The trend in our data does, however, not provide conclusive evidence regarding this matter.

With regard to the relationship between feedback processing and internal error detection, we can therefore conclude that although the electrophysiological data indicated clear neural responses to negative feedback and a developing indication of error detection, the current findings do not indicate a direct relation between FRN and ERN effects, but hint at a relation between P300 effects and the development of an ERN. In this respect, it may be added that the P300 effects in the present dataset were much stronger than the FRN effect, which may have affected the outcomes.

## Conclusions

In conclusion, the present findings show behavioral improvement following feedback, accompanied by signs of internal error detection based on representations stabilized by learning. The ERP results point to a discrepancy in CRN and ERN amplitudes that evolves over the course of a learning task following several rounds of feedback. In initial phases of learning, behavioral performance and the absence of a significant ERN effect indicated that the representations of L2 learners regarding a difficult grammatical feature were not strong enough to warrant detection of errors. Response-locked negativities for both correct and incorrect responses seem to indicate uncertainty, possibly due to unstable L2 representations in combination with competing L1 representations. Although further L2 learning studies should examine this more closely, the presence of a conflicting representation during memory retrieval could imply that errors are less easily detected. Following three rounds of feedback, the pattern of results showed that the formation of stable representations had at least been partially accomplished. We found evidence for internal error detection in the form of a small ERN, while the presence of robust FRN and P300 effects pointed to the importance of feedback throughout the learning task. Yet, the expected effect of the P300 as a predictor of subsequent behavioral learning was too small to draw firm conclusions. In line with present notions on performance monitoring, the results seem to indicate that external feedback leads to the development of internal error detection (see Wessel, 2012; Ullsperger et al., 2014; Hoffmann and Beste, 2015, for overviews of the different accounts). Important with respect to L2 acquisition is that L1 influences can complicate the process of error detection as long as feedback has not yet been internalized. All in all, the present findings indicate that L2 learning, as a realistic form of high-level learning, provides a useful method to examine neural mechanisms of underlying error monitoring in relation to memory based learning.

## Ethics statement

This study was carried out in accordance with the recommendations of the Ethics Committee of the Faculty of Social Sciences of Radboud University with written informed consent from all subjects, and complying with EU legislation.

## Author contributions

SB, KL, CD, and HB designed the experiment. SB prepared the materials and conducted the study. SB and CD analyzed the data. SB, KL, CD, and HB wrote the paper.

### Conflict of interest statement

The authors declare that the research was conducted in the absence of any commercial or financial relationships that could be construed as a potential conflict of interest.

## Footnotes

^1^Confusingly, in the L2 acquisition domain the term ‘mistake’ refers to performance errors, while ‘error’ denotes systematic errors (Corder, 1967).
