# Supplementary material for: Electrophysiological Correlates of Error Monitoring and Feedback Processing in Second Language Learning
Source: Front Hum Neurosci. 2017 Jan 30;11:29. doi: 10.3389/fnhum.2017.00029 (PMC5277024; doi:10.3389/fnhum.2017.00029)
Supplement: Supplementary file 1 [file DataSheet1.pdf]

APPENDIX A

Language learning motivation and anxiety questionnaire in German (with English translations)

1. Allgemeine Niederländisch Lernmotivation (general motivation)
    a. Niederländisch zu können ist mir wichtig.
       [*Knowing Dutch is important to me.*]
    b. Ich arbeite gern hart, um mein Niederländisch zu verbessern.
       [*I am prepared to work hard to improve my Dutch.*]
    c. Niederländisch zu lernen ist/ war eine Zeitverschwendung.
       [*Learning Dutch is/was like a waste of time.*]
    d. Ich habe Niederländisch nur gelernt, um dem Unterricht folgen zu können.
       [*I only learned Dutch to be able to follow classes.*]

2. Perfektionismus (perfectionism)
    a. Ich habe geringes Interesse daran, alle Aspekte des Niederländischen zu lernen.
       [*I have little desire to master all aspects of Dutch grammar.*]
    b. Ich achte auf feine Unterschiede zwischen Deutsch und Niederländisch.
       [*I pay attention to subtle differences and similarities between Dutch and German.*]
    c. Ich bin genervt, wenn ich unnötige Fehler im Niederländischen mache.
       [*I get annoyed when I make unnecessary mistakes in Dutch.*]
    d. Ich habe nicht das Ziel, Niederländisch perfekt zu sprechen.
       [*I do not aim to speak Dutch perfectly.*]

3. Angst die Fremdsprache zu nutzen (confidence)
    a. Es beunruhigt mich, wenn andere deutsche Studenten scheinbar besser Niederländisch sprechen als ich.
       [*It worries me that other German students in my classes seem to speak Dutch better than I do.*]
    b. Ich fühle mich sicher wenn ich Niederländisch spreche.
       [*I feel confident when speaking Dutch in any situation.*]
    c. Es ist mir peinlich, einen Fehler im Niederländischen zu machen.
       [*I feel embarrassed when I make a mistake in Dutch.*]
    d. Ich finde es total in Ordnung, wenn andere mein Niederländisch korrigieren.
       [*I feel at ease when other people correct my Dutch.*]

4. Ausdauer (perseverance)
    a. Wenn es zu schwierig ist etwas auf Niederländisch sagen, gebe ich schnell auf.
       [*If it becomes too difficult to say something in Dutch, I easily give up.*]
    b. Ich vermeide es Niederländisch zu sprechen wann immer möglich.
       [*I prefer to avoid speaking Dutch whenever I can.*]
    c. Ich achte auf meine Fehler und versuche von ihnen zu lernen.
       [*I pay attention to my own mistakes and try to learn from them.*]
    d. Ich frage Muttersprachler häufig nach der besten Art und Weise Dinge auf Niederländisch zu sagen.
       [*I ask native speakers detailed questions on how to say things in Dutch*]

Note: ratings were given on a 5-point scale ranging from fully agree to fully disagree and points were assigned depending on the wording (positive or negative) of the statement.

APPENDIX B

Stimulus materials in Dutch followed by German (and English) translations

**Target items**			
gender incongruent cognates			
de armband	*das Armband [bracelet]*	het adres	*die Adresse [address]*
de auto	*das Auto [car]*	het altaar	*der Altar [altar]*
de baby	*das Baby [baby]*	het anker	*der Anker [anchor]*
de boot	*das Boot [boat]*	het bedrag	*der Betrag [amount]*
de cello	*das Cello [cello]*	het blok	*der Block [block]*
de datum	*das Datum [date]*	het gordijn	*die Gardine [curtain]*
de foto	*das Foto [photograph]*	het hert	*der Hirsch [deer]*
de gevangenis	*das Gefängnis [jail]*	het kanon	*die Kanone [canon]*
de handdoek	*das Handtuch [towel]*	het kompas	*der Kompass [compass]*
de hoorn	*das Horn [horn]*	het krijt	*die Kreide [chalk]*
de kabel	*das Kabel [cable]*	het masker	*die Maske [mask]*
de kameel	*das Kamel [camel]*	het nummer	*die Nummer [number]*
de kano	*das Kanu [canoe]*	het orgel	*die Orgel [organ]*
de krokodil	*das Krokodil [crocodile]*	het palet	*die Palette [palette]*
de olie	*das Öl [oil]*	het paspoort	*der Pass [passport]*
de piano	*das Piano [piano]*	het pistool	*die Pistole [pistol]*
de pleister	*das Pflaster [band aid]*	het schrift	*die Schrift [writing system]*
de puzzel	*das Puzzle [puzzle]*	het schuim	*der Schaum [foam]*
de radio	*das Radio [radio]*	het spek	*der Speck [bacon]*
de sofa	*das Sofa [couch]*	het toilet	*die Toilette [toilet]*
de steak	*das Steak [steak]*	het uniform	*die Uniform [uniform]*
de taxi	*das Taxi [taxi]*	het verband	*der Verband [bandage]*
de telefoon	*das Telefon [telephone]*	het zand	*der Sand [sand]*
de zebra	*das Zebra [zebra]*	het zweet	*der Schweiss [sweat]*

**Filler items**			
	gender congruent cognates		
de banaan	*die Banane [banana]*	het bestek	*das Besteck [cutlery]*
de bloem	*die Blume [flower]*	het brood	*das Brot [bread]*
de boter	*die Butter [butter]*	het gewicht	*das Gewicht [weight]*
de hond	*der Hund [dog]*	het glas	*das Glas [glass]*
de schoen	*der Schuh [shoe]*	het nest	*das Nest [nest]*
de stoel	*der Stuhl [chair]*	het schaap	*das Schaf [sheep]*
de tomaat	*die Tomate [tomato]*	het zwaard	*das Schwert [sword]*
de vlieg	*die Fliege [fly]*	het vliegtuig	*das Flugzeug [airplane]*
	gender congruent non-cognates		
de envelop	*der Umschlag [envelope]*	de tafel	*der Tisch [table]*
de geit	*die Ziege [goat]*	het brein	*das Gehirn [brain]*
de kast	*der Schrank [wardrobe]*	het cadeau	*das Geschenk [present]*
de krant	*die Zeitung [newspaper]*	het fruit	*das Obst [fruit]*
de laars	*der Stiefel [boot]*	het ontbijt	*das Frühstück [breakfast]*
de peer	*die Birne [pear]*	het schilderij	*das Gemälde [painting]*
de riem	*der Gürtel [belt]*	het varken	*das Schwein [pig]*
de slagroom	*die Sahne [cream]*	het wiel	*das Rad [wheel]*
	gender incongruent non-cognates		
de bagage	*das Gepäck [luggage]*	de zeef	*das Sieb [sieve]*
de bioscoop	*das Kino [cinema]*	het blik	*die Dose [can]*
de fiets	*das Fahrrad [bicycle]*	het boeket	*der Strauss [bouquet]*
de groente	*das Gemüse [vegetable]*	het bord	*der Teller [plate]*
de jurk	*das Kleid [dress]*	het fornuis	*der Herd [stove]*
de kip	*das Huhn [chicken]*	het potlood	*der Bleistift [pencil]*
de plank	*das Brett [shelf]*	het sieraad	*der Schmuck [jewellery]*
de tent	*das Zelt [tent]*	het stoplicht	*die Ampel [traffic light]*
